# From collocations to call-ocations: using linguistic methods to quantify animal call combinations

**DOI:** 10.1007/s00265-022-03224-3

**Published:** 2022-08-22

**Authors:** Alexandra B. Bosshard, Maël Leroux, Nicholas A. Lester, Balthasar Bickel, Sabine Stoll, Simon W. Townsend

**Affiliations:** 1 Department of Comparative Language Science, University of Zurich, Affolternstrasse 56, 8050 Zurich, Switzerland; 2 Center for the Interdisciplinary Study of Language Evolution (ISLE), University of Zurich, Zurich, Switzerland; 3 Department of Psychology, University of Warwick, Coventry, UK

**Keywords:** Call combinations, Collocation analysis, Comparative approach, Non-random structure, Syntax

## Abstract

**Abstract:**

Emerging data in a range of non-human animal species have highlighted a latent ability to combine certain pre-existing calls together into larger structures. Currently, however, the quantification of context-specific call combinations has received less attention. This is problematic because animal calls can co-occur with one another simply through chance alone. One common approach applied in language sciences to identify recurrent word combinations is collocation analysis. Through comparing the co-occurrence of two words with how each word combines with other words within a corpus, collocation analysis can highlight above chance, two-word combinations. Here, we demonstrate how this approach can also be applied to non-human animal signal sequences by implementing it on artificially generated data sets of call combinations. We argue collocation analysis represents a promising tool for identifying non-random, communicatively relevant call combinations and, more generally, signal sequences, in animals.

**Significance statement:**

Assessing the propensity for animals to combine calls provides important comparative insights into the complexity of animal vocal systems and the selective pressures such systems have been exposed to. Currently, however, the objective quantification of context-specific call combinations has received less attention. Here we introduce an approach commonly applied in corpus linguistics, namely collocation analysis, and show how this method can be put to use for identifying call combinations more systematically. Through implementing the same objective method, so-called call-ocations, we hope researchers will be able to make more meaningful comparisons regarding animal signal sequencing abilities both within and across systems.

**Supplementary Information:**

The online version contains supplementary material available at 10.1007/s00265-022-03224-3.

## Introduction

Over the last 20 years, there has been a growing interest into the combinatorial abilities of animals, namely the propensity to sequence context-specific calls (i.e. meaning-bearing units, see Suzuki and Zuberbühler 2019) into larger potentially meaningful structures (Arnold and Zuberbühler 2006; Ouattara et al. 2009; Engesser et al. 2016; Suzuki et al. 2016; Collier et al. 2020). Combinatoriality is one mechanism that can increase the expressive potential of a finite vocal repertoire (Nowak et al. 2000). Data on the combinatorial capacities of non-human animals therefore provide important comparative insights into the complexity of animal communication systems and the selective pressures such systems have been exposed to (Collier et al. 2020). Data on combinatoriality also hold great promise in furthering our understanding of the similarities between animal communication and human language given that, for many years, it was assumed that the systematic combination of meaning-bearing units (i.e. syntax) was a phenomenon unique to language (Hurford 2012). Emerging examples of meaning-bearing syntactic-like structures in non-human primates and non-primate animals suggest this particular assumption was indeed premature (Arnold and Zuberbühler 2006; Coye et al. 2015, 2016; Engesser et al. 2016; Suzuki et al. 2016; Berthet et al. 2019; Collier et al. 2020), and such data even have the potential to further our understanding of the evolutionary progression of our own communication system (Townsend et al. 2018; Leroux and Townsend 2020).

In light of the communicative and evolutionary insights that research on call combinations can provide, it is surprising that, in contrast to a rich literature on sequential dynamics in songs (Honda and Okanoya 1999; Suzuki et al. 2006; Kershenbaum et al. 2014; Kershenbaum and Garland 2015; Sainburg et al. 2019, and for a review of the topic: ten Cate and Okanoya 2012), to date, objective means of capturing the statistical association patterns in context-specific call combinations have remained less explored. This is problematic as animal calls may occur in rapid succession, representing mere read-outs of contextual shifts. A method to capture greater-than-chance co-occurrence of calls is therefore central to reliably detect and identify non-random (i.e. potentially relevant) animal call combinations.

Similar methodological issues have been encountered in research on language learning and use (Bartsch 2004; Evert 2008; Gries 2013; Gablasova et al. 2017). One approach frequently implemented to identify combinations of words (mostly bigrams, i.e. two-word/two-call structures) in large written and spoken corpora is collocation analysis (for a review, see Gries 2013). Collocation analyses can take several forms, but the core commonality is that it contrasts the frequency with which specific words combine to measure the relative exclusivity of their relationship within a corpus (Church et al. 1991; Kennedy 1991; Gries and Stefanowitsch 2004; Nesselhauf 2005). In other words, such analyses reveal whether particular word/call combinations are more common than would be expected given an assumed random baseline (e.g. the uniform distribution, in which each combination is equally likely). For example, in English “*drink*” collocates with “*coffee*” and “*going*” collocates with “*to*” (to form the future tense or describe a motion event). Thus, collocation analyses can be understood as a simple yet highly informative measure of the influence a lexical item has on its neighbours. Collocation analyses differ from other related conditional probability measurements that capture the distributions over vectors, such as Markov chains, since Markov chains are used to primarily model the running dynamics of all relationships between elements by calculating the probabilities over entire sequences, whilst collocation analyses take a distributional matrix as input to describe the specific relationship between two predetermined elements based on how they relate to a baseline (calculated via a statistic such as the hypergeometric probability or via mutual information estimates, e.g. Stefanowitsch and Gries 2003; Bartsch 2004; Xiao and Mcenery 2006; Lehecka 2015). Therefore, in situations when combinations of signals do not exceed the size of two and where the core goal is to probe whether these binary associations are produced above chance, collocation analysis is a suitable tool.

In this paper we highlight that considering animal context-specific call data in a similar way as to how language data are treated (i.e. as a corpus) affords the unique opportunity to apply a variety of analytical tools habitually implemented in language sciences to study similar questions (see also Berwick et al. 2011; Schlenker et al. 2016a, b). Specifically, we demonstrate the application of collocation analyses to empirically identify combinations of two calls, henceforth termed *bigrams*, in a suite of variable, synthetically created non-human animal data sets, and the relative merits of doing so. We focus on two specific forms of collocational measurements commonly implemented in language sciences: Multiple Distinctive and Mutual Information Collocation Analyses (Gries 2014).

## Collocation analyses—Multiple Distinctive and Mutual Information approaches:

Multiple Distinctive Collocation Analysis (MDCA) is primarily used when investigating and testing what meaning-bearing units collocate with what grammatical constructions (collostructional analysis). In addition to statistically contrasting all possible bigram combinations to estimate whether a given bigram occurs at frequencies higher or lower than what would be expected by chance (Gries and Stefanowitsch 2004; Hilpert 2006), the output of MDCA also provides a superficial estimate of bigram ordering, namely whether the combination is sensitive to the position of the calls comprising it (e.g. is A-B as frequent as B-A). MDCA uses multiple (corrected) binomial probability statistics over the cross-tabulated frequencies of word pairs in the sample (this approach serves as an approximation of multinomial probability values, which allow the comparison of an element with more than two alternatives; see Gries and Stefanowitsch 2004; Gries 2014). Importantly, since an MDCA is calculated via the binomial probability mass function, it is not constrained by the usual sampling assumptions, making it suitable for skewed, non-random, and small corpora of the kind often encountered in animal communication (Gries and Stefanowitsch 2004; Hilpert 2006; Gries 2014).

One recurrent issue for the analysis of linguistic corpora is the fact that any corpus represents an incomplete—or undersampled—representation of the target linguistic system—i.e. some two-word combinations can be under-represented or even absent from a corpus when their “true” probability of occurrence is higher (note that this also affects all other probabilities in the sample, which are artificially inflated, because other probabilities are underestimated). Such undersampling leads to misleading estimates of the significance of certain bigrams in the corpus (Gries and Stefanowitsch 2004; Hilpert 2006). Mutual Information Collocation Analysis (MICA) however actually overestimates low-frequency values (Church and Hanks 1990; Evert 2005), which can be an advantage in animal communication corpora, as low-frequency pairings, a common feature of non-human vocal data sets, are not overlooked but flagged as potential combinatorial candidates. MICA calculates the variability of two predetermined co-occurring items through computing information values via observed frequency divided by expected frequency (i.e. baseline). Specifically, MICA (when expressed in binary logarithms) gives the bits of information that are shared between two distributions. In the case of collocations, this shared information can be construed as degree and kind of association (meaning the size of the collocational strength number and whether elements attract or repulse each other). MICA is computed as the ${log}_{2}$ of the ratio of frequency of observed sequences $({e}_{i}, {e}_{i+1}$; where e = element and i = position) over the expected frequency $\mathrm{E}({e}_{i},{e}_{i+1}$), where $\mathrm{E}({e}_{i},{e}_{i+1}$) is expressed as the product of the frequencies of $\frac{{e}_{i}, {e}_{i+1} }{sample size}$. A MICA score of 0 means that the two distributions vary independently. MICA scores > 0 indicate greater-than-expected rates of co-occurrence. MICA scores < 0 indicate less-than-expected rates of co-occurrence. This measure thus differs from MDCA, which uses binomial probability values over a $2\times 2$ frequency matrix.

In the remainder of the paper, we apply both forms of collocation analyses using an existing R script provided by Stefan Gries (2014, see supplementary) to four artificially created pseudo data sets.

### Call combinations in artificial data sets: a guide

We first constructed a repertoire broadly consistent in size with the known repertoires of other various primate species (Boesch and Crockford 2005; Ouattara et al. 2009; Leroux et al. 2021). Specifically, ten hypothetical call types ranging from tonal whistles and twitters to noisy barks and coughs were used (see Table 1 and appendix I for the exact distribution of the data). From this repertoire we then built four artificial sets of call combination distributions. Since most research in context-specific call systems has detected two-call combinations (e.g. Arnold and Zuberbühler 2006; Coye et al. 2015; Engesser et al. 2016; Suzuki et al. 2016; Collier et al. 2020), we constrained our data sets to only include instances in which two calls from the repertoire co-occurred with each other to form bigrams (see appendix V for the raw input files).

**Table 1:** Distribution of identified bigrams occurring in the four artificial vocal repertoires split along the two variables i) size of data set and ii) extent of recombination. The last two rows show the number of combinations that are described in the data set (combinations) and, therefore, how many calls a data set comprises (data set size). See appendix I for detailed distribution. The values are larger for the recombinational data sets as in these data sets all call types recombine with other call types outside of the three call combinations of interest (for details see appendix I)

	*SE*	*SR*	*LE*	*LR*
	*Small Exclusive*	*Small Recombination*	*Large Exclusive*	*Large Recombination*
*Huff-Puff*	4	4	40	40
*Peep-Howl*	5	5	50	50
*Howl-Peep*	7	7	70	70
*Combinations*	16	49	160	490
*Data set size*	32 calls	98 calls	320 calls	980 calls

Although in this paper we use artificial data sets, it is important to note that in order to initially acquire such data from captive or free-living animals, researchers should ideally collect focal data (Altmann 1974), noting the raw number of times context-specific calls from the repertoire combine with each other (with a combination often being defined as two calls being temporally closely positioned, see, for example, Boesch and Crockford 2005; Coye et al. 2015; Collier et al. 2017). This will allow the generation of a frequency matrix on which collocation analysis can then be applied (for an example of such a matrix, see appendix I).

To showcase the advantages and disadvantages of MDCA and MICA respectively, we guided the simulation of the data sets along multiple distributional variables. Firstly, the combinatorial data sets were created according to two dimensions important to consider when identifying call combinations, namely the size of the data set, including the number of times the target combinations are detected, and the recombinatorial patterns observed, i.e. if the different call types recombine with only a few or with many other call types in the repertoire. Specifically, we created synthetic vocal data sets of i) small (i.e. S) and large size (i.e. L) and ii) data sets designed with call types that appear in combination with only one other call type exclusively (i.e. E) or that recombine with many different call types (i.e. R) resulting in four distinct data sets: small-exclusive (SE), large-exclusive (LE), small-recombinatorial (SR) and large-recombinatorial (LR). Secondly, the emergent two-call combinations of interest in the four synthetic data sets were created to be i) either stereotyped or flexible in call order (e.g. either appeared in a specific order or had no linearisation pattern) and to have ii) a varying frequency within the data sets, i.e. one pair (Huff-Puff) appearing at a very low frequency level in comparison to other higher frequency pairings (Howl-Peep/Peep-Howl). To capture these relationships, we applied both MDCA and MICA to our data sets.

Since we generated a variety of call associations along these dimensions, we expected collocation analyses to reveal at least some significant relationships between calls. More precisely, we predicted that the MDCA values would increase positively with increased data set size and that the analyses would reveal if a bigram pair is prone to a specific order. We also predicted that based on the way MICA is calculated, the analysis would be more resilient to sample size differences and highlight the low-frequency bigrams more strongly than MDCA. Lastly, we expected both analyses to show weaker collocation strengths once calls from a candidate combination recombine with other calls in the repertoire.

### Multiple Distinctive Collocation Analysis:

In a first step, we applied a Multiple Distinctive Collocation Analysis, where call dependencies within bigrams were calculated for each data set using binomial probability values on each possible bigram combination (Gries and Stefanowitsch 2004; Hilpert 2006; Gries 2014). Specifically, the binomial probability mass function renders probability values for each word/call with each other in each construction, which are then log-transformed for spacing and make it possible to estimate whether a given bigram occurs less (negative “pbins”, Table 2) or more (positive “pbins”, Table 2) than what would be expected by chance. In addition, the values (used here explicitly, since they reflect the relative association of calls, but whilst simultaneously accounting for sample size) correspond to p estimation significance levels (i.e. pbin > 3: P < 0.001, > 2: P < 0.01, > 1.3: P < 0.05, if < 1.3: NS). Since the aim here is to identify potential candidates for meaningful call combinations, we will focus only on positive values that highlight an attraction between two call types. For positive pbin values, the higher the value for two calls, the greater their collocational strength.

**Table 2:**
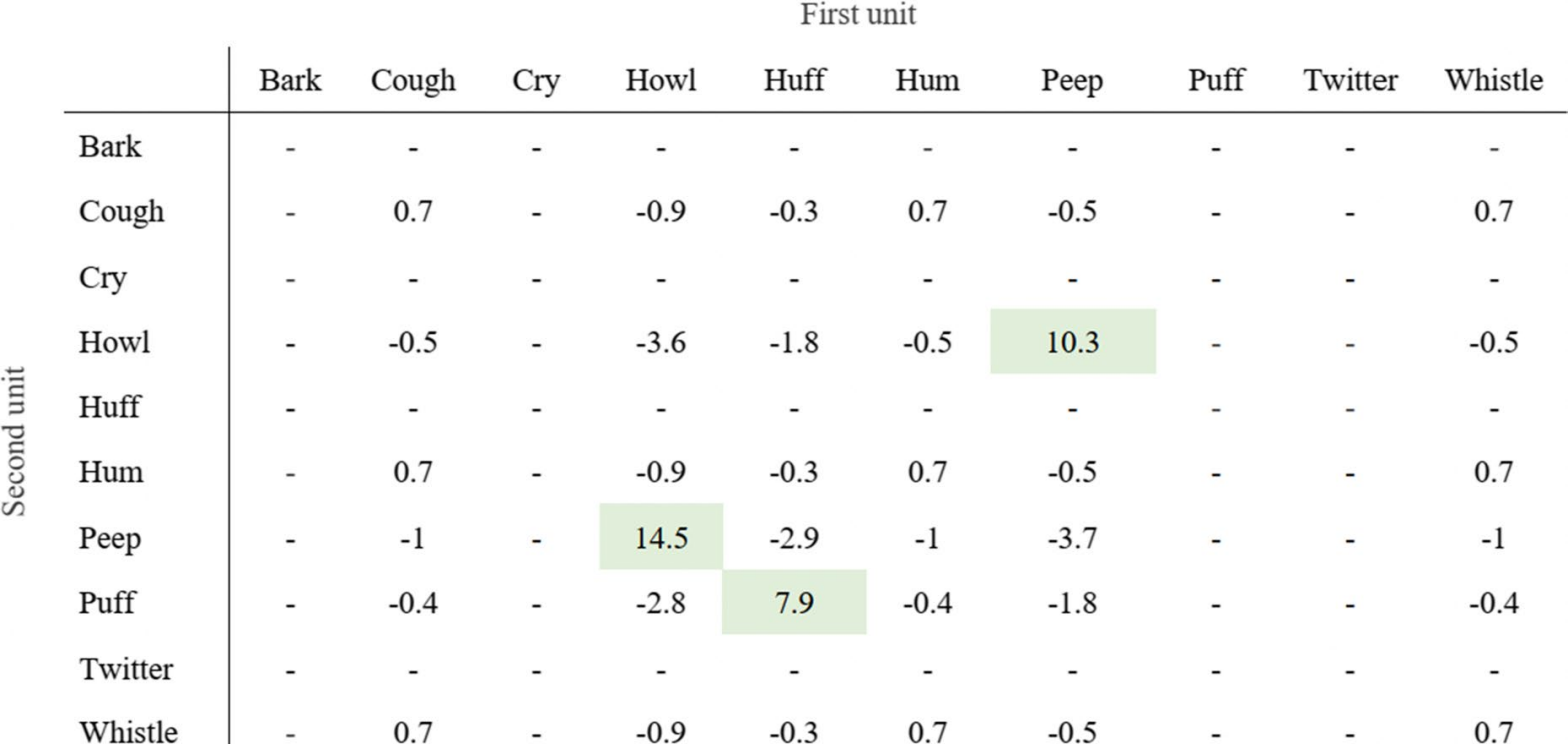
Example of a Multiple Distinctive Collocation Analysis output for the bigrams of one of the four artificial vocal data sets (large-recombinations). Columns and rows show the first and second unit within a call combination, respectively. Values are pbins and can be translated to p estimation values (abs(pbin) > 3: P < 0.001, > 2: P < 0.01, > 1.3: P < 0.05). Relevant combinations are coloured in green

Table 3 shows the results of the Multiple Distinctive Collocation Analysis applied to all four synthetic data sets. Comparing the collocational strength of the combinations of interest across the four data sets indicates larger collocational values are associated with larger data sets. For example, the pbin value (i.e. collocational strength) for the Huff-Puff combination is 2.4 in the small-exclusive data set (SE) whilst it is multiplied by 10 (24.1) in the large-exclusive data set (LE). Furthermore, collocational strengths decrease as a result of recombination in the data sets: the more exclusive a combination, the stronger the collocational strength. For example, in a small data set, the Huff-Puff combination’s collocational strength is only 1.2 when the calls occurred with other call types in the data set (SR), while this value doubles (2.4) when the two calls occur exclusively with each other (SE) (see Table 3).

**Table 3:** Multiple Distinctive Collocation values for the bigrams in the four artificial vocal data sets. SE (small-exclusive), SR (small-recombinations), LE (large-exclusive), and LR (large-recombinations). Values are pbins and can be translated to p estimation values (abs(pbin) > 3: P < 0.001, > 2: P < 0.01, > 1.3: P < 0.05)

	*Multiple Distinctive Collocation Analysis*			
	*SE*	*SR*	*LE*	*LR*
*Huff-Puff*	2.4	1.2	24.1	7.9
*Peep-Howl*	2.5	1.4	25.3	10.3
*Howl-Peep*	2.5	1.9	25.1	14.5

One additional feature of MDCA is that it tests whether the calls appear in a specific order (linearisation). The combination of Huff-Puff showed a notable attraction between the two constituent calls in this specific order, given that it solely occurred in this order. However, Peeps and Howls occurred more fluidly with each other, and therefore displayed a high level of attraction no matter the linearisation (Peep-Howl and Howl-Peep) suggesting that either order did not matter for this particular combination, or that Peep and Howl form two significant, differently ordered, bigrams.

### Mutual Information Collocation Analysis:

In light of the aforementioned advantages associated with an information-based approach, we complemented the MDCA analysis by also running MICA on the synthetic data sets. As previously mentioned, MICA calculates the collocational strength of a specific call type with every other call type it collocates with. To do so, the *joint observed frequency* of a specific bigram is divided by its *joint expected frequency* and then  ${log}_{10}$ transformed*.* Concretely, the number of times the calls actually appear in combination is divided by the number of times the calls would appear in combination if every call was randomly distributed throughout the data set. Once more, the higher the collocation value, the stronger the collocational strength between two units (again, we focus on positive values that indicate an attraction only, see Table 4). As with MDCA, pbins represent the ${log}_{10}$ transformed values as a way of highlighting that highly positive pbin values stand for larger collocational strength between calls/words (i.e. if the absolute value of pbin > 3: P < 0.001, > 2: P < 0.01, > 1.3: P < 0.05, if < 1.3: NA).

**Table 4:**
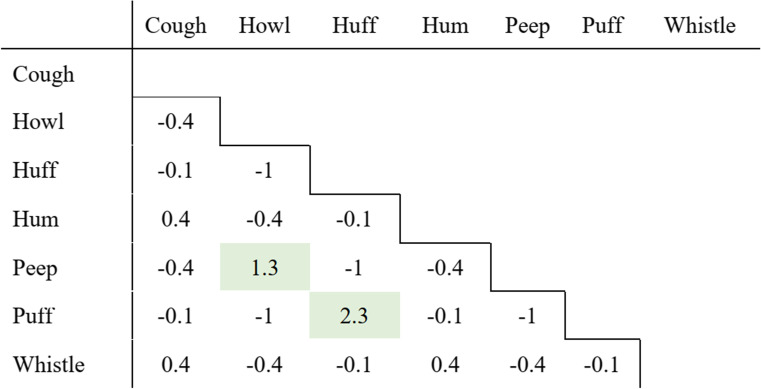
Example of a Mutual Information Collocation Analysis output for the bigrams of one of the four artificial vocal data sets (large-recombinations). Columns and rows show the first and second unit within a call combination respectively. Values are pbins and can be translated to p estimation values (abs(pbin) > 3: P < 0.001, > 2: P < 0.01, > 1.3: P < 0.05). Relevant combinations are coloured in green

Not accounting for any specific ordering of the structures in the analysed input, the Mutual Information Collocation Analysis demonstrated a relative attraction within two bigrams (Huff-Puff and Peep-Howl/Howl-Peep see Table 5). Furthermore, due to the way MICA is calculated, its collocational values are not affected by sample size. However, they are influenced by the frequency distribution of the call combinations within the sample. Specifically, MICA only highlights bigrams for call types that, firstly, appear exclusively in combination with their collocational partner (low level of recombination) and, secondly, those that occur less frequently in the data set in general (low frequency pairings). This means that the distributions of the call combinations have an impact on each other’s values: the collocational value for Huff-Puff is larger than the value for Peep-Howl as Huff-Puff only appears 4 or 40 times in the data sets, while Peep-Howl appears 12 or 120 times in the data sets respectively. Thus, MICA provides high values for very low-frequency pairings, in this case Huff-Puff.

**Table 5:** Mutual Information Collocation values for the bigrams in the four artificial vocal data sets. SE (small-exclusive), SR (small-recombinations), LE (large-exclusive), LR (large-recombinations). Values are pbins and can be translated to p estimation values (abs(pbin) > 3: P < 0.001, > 2: P < 0.01, > 1.3: P < 0.05). As here, MICA does not control for specific ordering of calls in a structure, the bigrams Howl-Peep and Peep-Howl are considered to be the same combination, rendering only one entry, namely *Peep-Howl,* that incorporates both bigrams

	*Mutual Information Collocation Analysis*			
	*SE*	*SR*	*LE*	*LE*
*Huff-Puff*	3	2.3	3	2.3
*Peep-Howl*	1.4	1.3	1.4	1.3

## Discussion

When conceptualising animal vocal data in the same way as a language corpus, we show that methods habitually implemented in corpus linguistics to identify *word combinations—*namely collocation analyses*—*can be transferred reliably to non-human signal sequences to highlight *call combinations* that are promising for further investigation. Specifically, collocation analyses help disentangle “true”, or non-random, call combinations from happenstance juxtapositions of single calls. This is critical when investigating potentially meaningful structuring within animal signal sequences.

We have described two collocational measurements—Multiple Distinctive and Mutual Information Collocation analysis—and highlighted the advantages when applying these respective analyses to synthetically created data sets. Both measurements identify call combinations in the data, however, as Table 6 illustrates, there are some key differences. For example, MDCA’s collocation values show a scalar increase in line with the factor the sample size was increased with, while MICA’s values, since they are based on joint probability, are insensitive to sample size (except for precision increasing with increasing corpus size).

**Table 6:** Comparison of Multiple Distinctive Collocation Analysis and Mutual Information Collocation Analysis. Arrows represent if the collocation values get lower or higher due to a characteristic of the data set (small data set) or a call combination (recombination, low frequency, linearisation). The checkmark indicates that MDCA identifies ordering patterns, while MICA does not. *NA* designates that the size of the data set has no effect on MICA

*Characteristics of data input*					
		*Small data set*	*Recombination*	*Low frequency*	*Linearisation*
*Collocation analysis*	*MDCA*	↓	↓	↓	
	*MICA*	NA	↓	↑	X

Furthermore, an advantage of MDCA in particular is that it allows an estimate of the ordering of call combinations, a feature not present in MICA. Identifying variation in the temporal organisation of calls is necessary to design experiments probing the role of order on meaning. Results from the provided data sets indicate that not all of the identified combinations are characterised by ordering, a finding that is replicated when applying collocation analysis to real-world data sets (see Bosshard 2020; Leroux et al. 2021). For example, in chimpanzees, long distance pant-hoot vocalisations are collocated with food calls above chance level (pant-hoot + food call combination). The reversed order, however, did not show a high collocational attraction, implying that linearisation might be an important feature for this combination. This preliminary identification of call order therefore might serve as one possible additional filter when deciding which of the combinations detected from an animal data set to follow-up from an experimental perspective.

Of particular relevance is the fact that the collocational analyses applied here, especially MICA, were also sensitive to bigrams even when they occurred very infrequently in the data set. This is because collocational analyses consider the exclusivity of the combinatorial relationship: if calls combine extremely rarely, they will still be detected as long as their relationship together is exclusive (see Table 6). Since considerable variation underlies the frequency with which different call combinations occur in animal vocal systems (e.g. alarm call combinations are less frequent than social call combinations (Boesch and Crockford 2005; Collier et al. 2017; Leroux et al. 2022), we can be confident that collocational analysis will identify possibly relevant combinations, both common and rare.

Since MDCA and MICA have their own respective advantages and shortcomings, we advocate applying both MDCA and MICA to data sets and comparing the results of these analyses. As previously mentioned, MDCA, for example, is sensitive to sample size and to the recombination of calls and, particularly if there is considerable recombination in a data set, it will give more weight to call combinations that appear more frequently. MICA, on the other hand, whilst also sensitive to recombination of calls, is affected by internal call distribution, as it highlights combinations that are extremely low in frequency in comparison to other higher frequency combinations. Applying both analyses increases the probability that researchers isolate likely communicatively relevant combinations and ultimately makes comparisons across data sets more robust.

Whilst collocation analysis represents an important first step to isolate candidate signal combinations, follow-up systematic behavioural observations and perception experiments are still critical to further unpacking the putative function and meaning of such combinations. This may not be feasible for all combinations detected, though priority could be given to the ones with the strongest collocational association over both analyses, thus likely representing the most robust combination.

It is also important to note that this is not the first attempt to estimate the combinatorial nature of animal vocal systems, indeed other systematic approaches to capture the sequential dynamics and internal structuring of animal vocal sequences, specifically song, have been applied. Song structures generally comprise multiple elements and summarising relationships between these elements requires calculating the probability of entire sequences. In these instances, modelling-based approaches, including different Markovian and non-Markovian chain modellings (Suzuki et al. 2006; Briefer et al. 2010; Jin and Kozhevnikov 2011; Kershenbaum et al. 2014; Kershenbaum and Garland 2015; Sainburget al. 2019) are most appropriate.

Whilst in principle such modelling approaches (e.g. Markov chains) could be applied to context-specific call systems, collocation analysis affords the following advantages. Firstly, collocation analyses are easily applied across systems and provide a convenient and descriptive account of the combinatorial dependencies between vocal units (Firth 1957; Evert 2005, 2008). Secondly, call combinations in animals are arguably simplistic and rarely exceed the level of the bigram (two call combinations) for which collocation analysis is precisely designed. Although optimised for detecting bigrams, collocation analysis must not be restricted to combinations of context specific calls but can also be applied to explore relevant 2-element combinations in larger structures such as songs.

We argue that this approach outlined here represents a novel application of an objective method to quantify combinatorial features of animal communication systems. Although we focus here on vocal signals, collocation analysis can also be implemented in a variety of settings or contexts such as identifying other signal combinations from both the same and different modalities.

We hope that collocation analyses will be applied by other researchers in the field of animal communication as an additional way to disambiguate random from non-random combinatorial structures, whether that be the combination of meaning-bearing elements (vocal, non-vocal or multimodal) or pinpointing relevant bigrams present in longer sequences, e.g. songs. Future work could also build on these initial approaches through (i) improving collocation estimates through better accounting for uncertainty (e.g. in a Bayesian framework) and (ii) applying other, as yet unexplored, sequence-based methods currently used in language sciences to animal corpora as a means to answer further questions (e.g. skip-gram modelling for analysing rarer, larger call sequences, see Guthrie et al. 2006).

Ultimately, implementing the same objective, standardised methods such as collocation analysis could allow researchers to make more meaningful comparisons both within and across systems, at the individual, group, population or even species level.

## Supplementary Information

Below is the link to the electronic supplementary material.

Supplementary file1 (DOCX 1.39 MB)

## Data Availability

see supplementary material: research_data_artificial_dataset.

## Code availability

see Appendix I: R Code.
