# Supplementary material for: From collocations to call-ocations: using linguistic methods to quantify animal call combinations
Source: Behav Ecol Sociobiol. 2022 Aug 22;76(9):122. doi: 10.1007/s00265-022-03224-3 (PMC9395491; doi:10.1007/s00265-022-03224-3)
Supplement: Supplementary file 1 — Supplementary file1 (DOCX 1.39 MB) [file 265_2022_3224_MOESM1_ESM.docx]

From collocations to call-ocations: using linguistic methods to quantify animal call combinations

Behavioral Ecology and Sociobiology

**Supplementary Material**

Alexandra B. Bosshard^1,2*^, Maël Leroux^1,2^, Nicholas A. Lester^1,2^, Balthasar Bickel^1,2^, Sabine Stoll^1,2^ & Simon W. Townsend^1,2,3^

^1^Department of Comparative Language Science, University of Zurich, Switzerland

^2^Center for the Interdisciplinary Study of Language Evolution (ISLE), University of Zurich

^3^Department of Psychology, University of Warwick, UK

*Corresponding author:

A. B. Bosshard, Department of Comparative Language Science, University of Zurich, Thurgauerstrasse 30, 8050 Zurich, Switzerland.

*E-mail Address*: alexandra.bosshard@uzh.ch

**Appendix I: Synthetic data sets**

Table 1: SE (small-exclusive) data set:


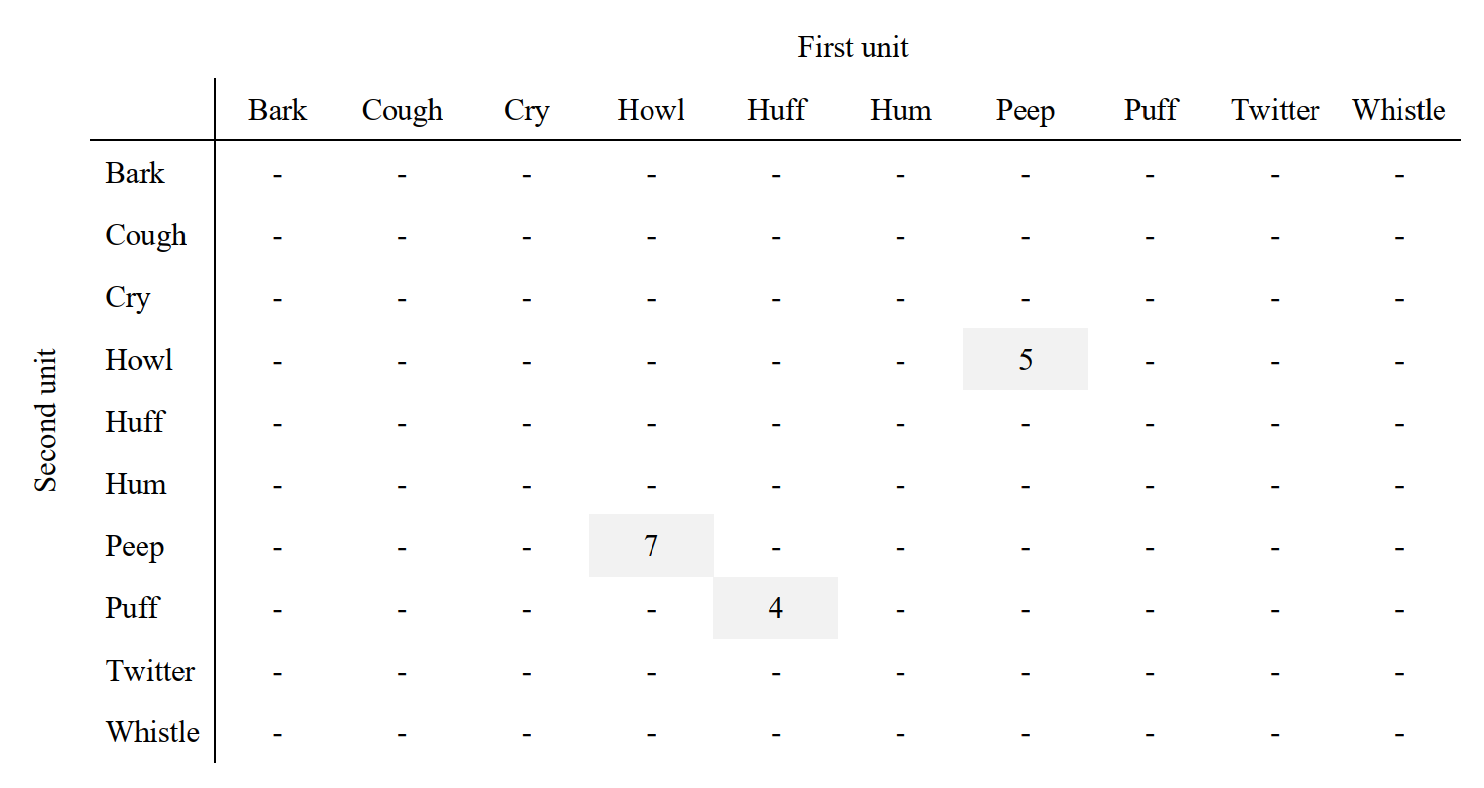


Table 2: SR (small-recombinations) data set:

For recombination, the call types Cough, Howl, Huff, Hum, Peep and Twitter were chosen to recombine with each other.


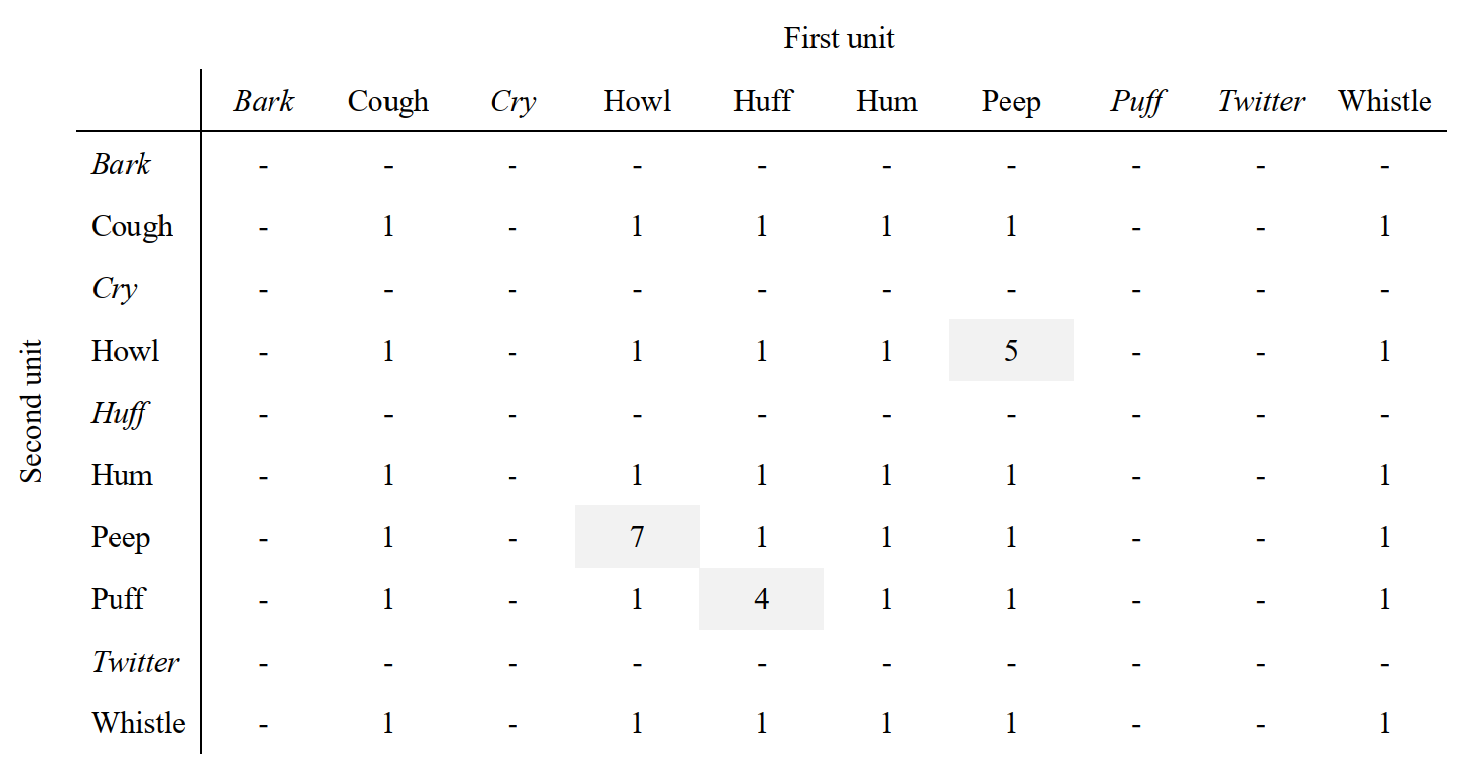


Table 3: LE (large-exclusive) data set:


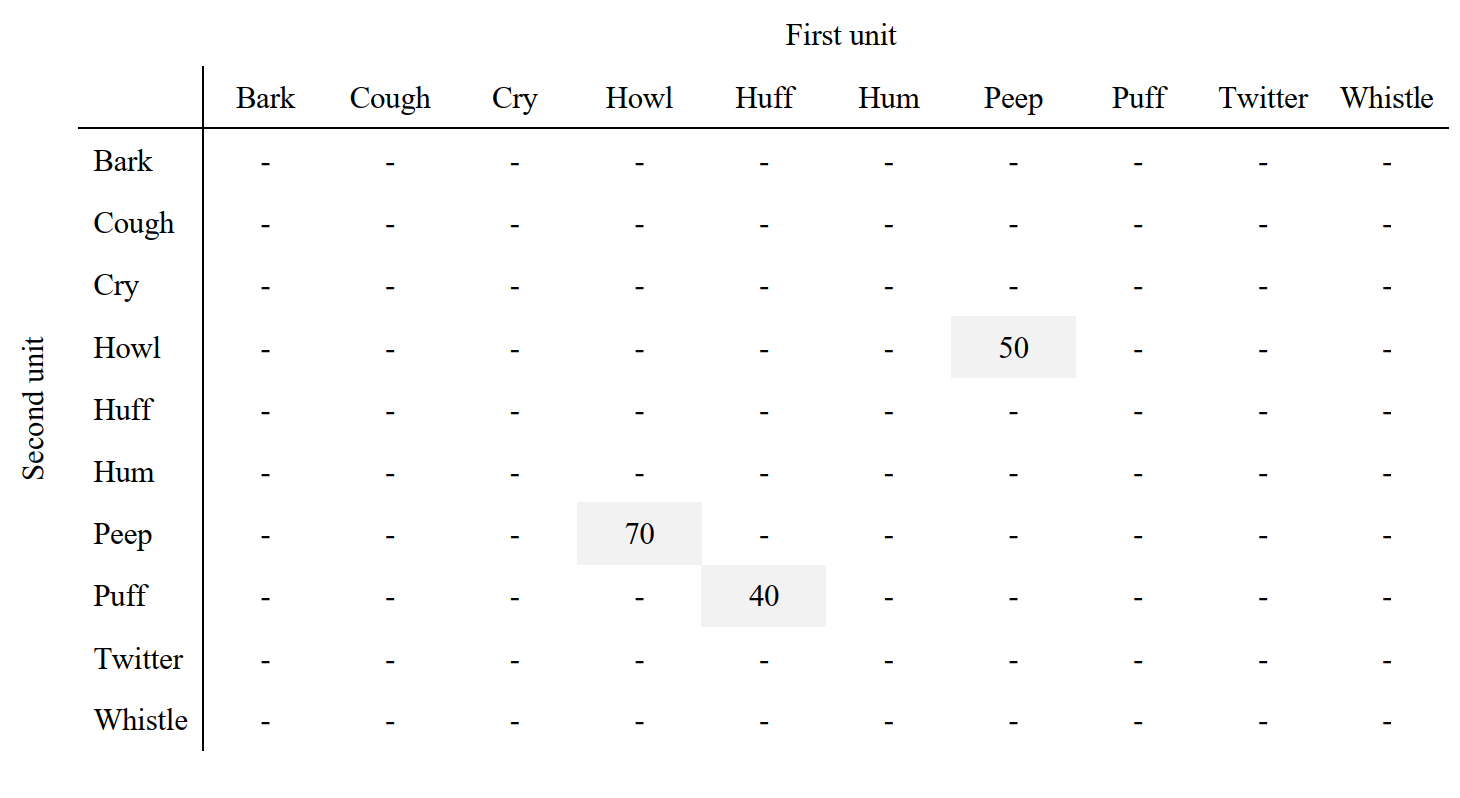


Table 4 : LR (large-recombinations) data set:

For recombination, the call types Cough, Howl, Huff, Hum, Peep and Twitter were chosen to recombine with each other.


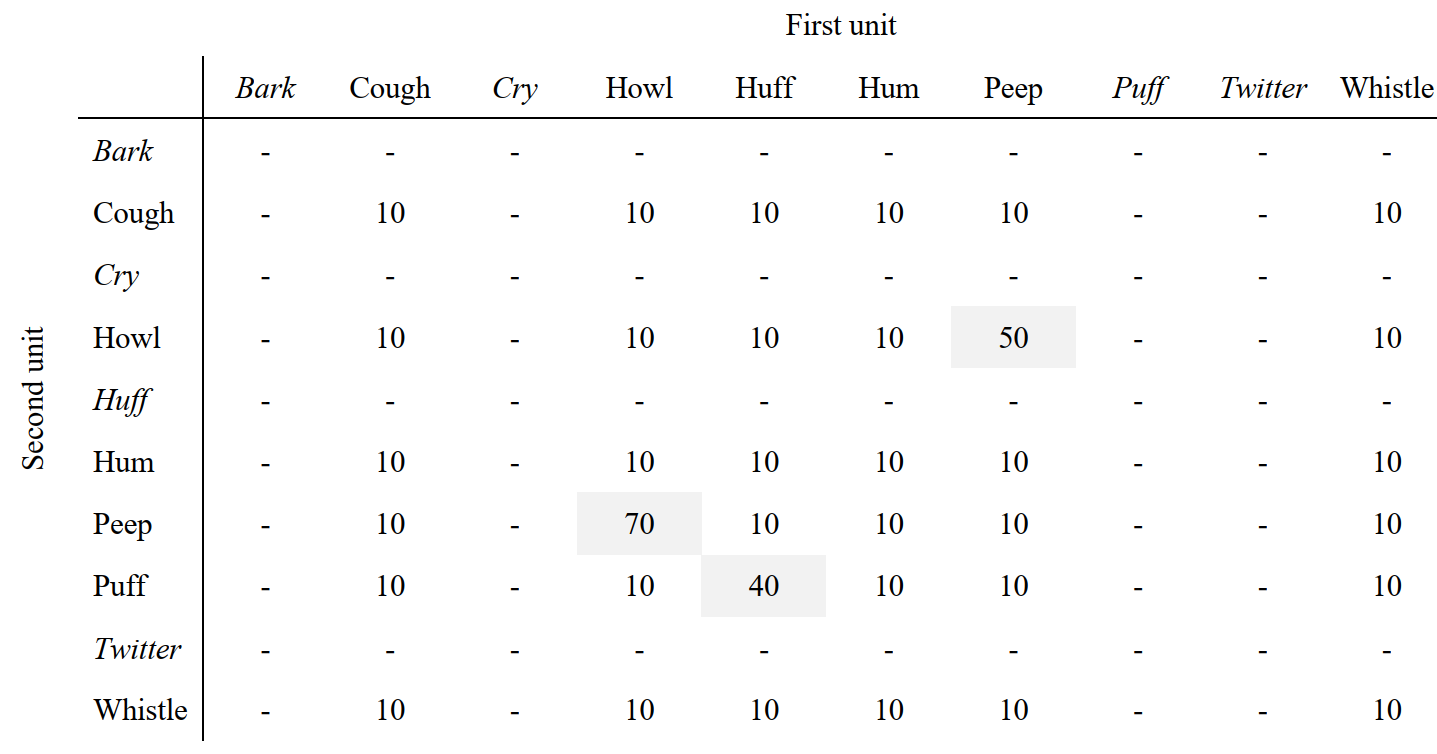


**
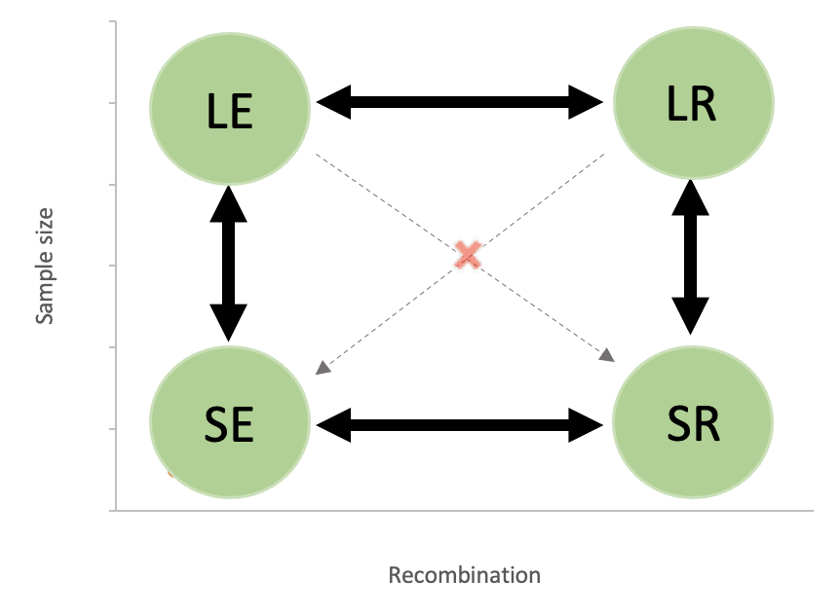
**

Figure 1: Dimensions of comparison between the data sets

We created the four data sets along two distributional variables; recombination of calls with other calls (x-axis) and sample size (y-axis). As this figure below shows, we then assessed and compared the collocational output along the black arrows (e.g., the large-exclusive with the large-recombinational data set). This meant that we only compared data sets that varied one variable at a time.

Table 5: Dimensions of comparison between the target combinations

As illustrated in the below table, target combinations differed according to two variables, one structural variable (if there is a specific order the calls appear in: linearisation) and one distributional variable (low or high frequency presence in the data sets).

|  | **Huff-Puff** | **Howl-Peep** | **Peep-Howl** |
| --- | --- | --- | --- |
| **Linearisation** | Yes | NA | |
| **Frequency** | Low | High | |

**Appendix II: MDCA tables**


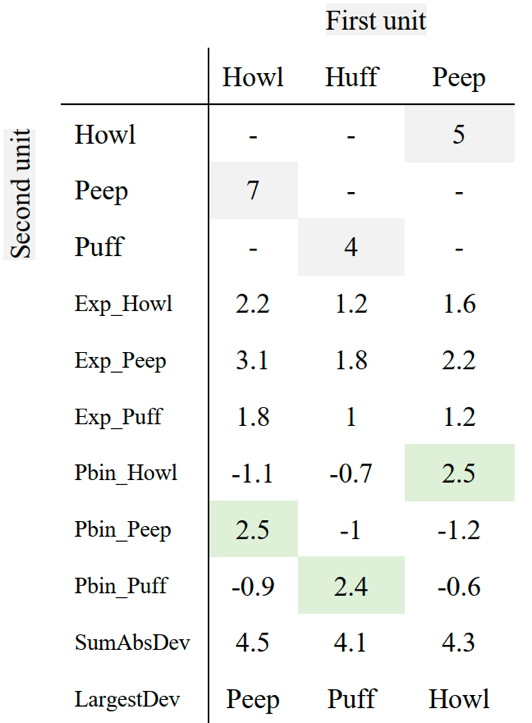
Table 6: SE (small-exclusive) data set MDCA matrix:

Table 7: SR (small-recombinations) data set MDCA matrix:


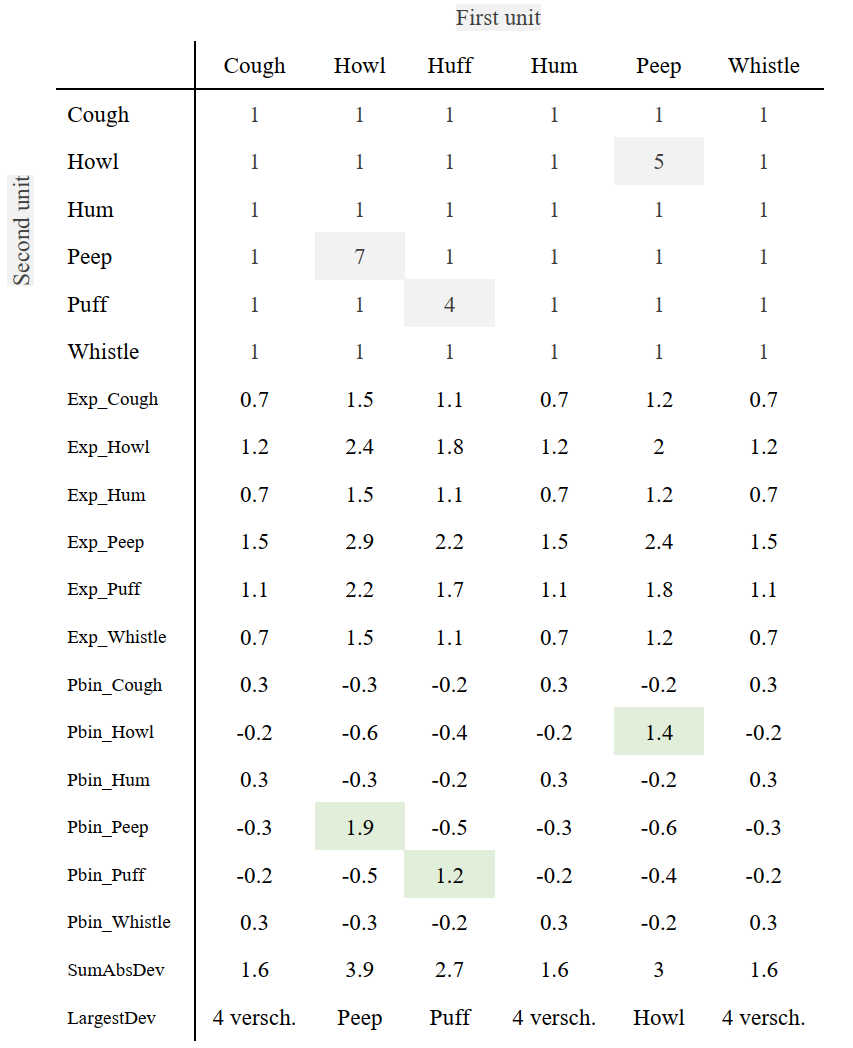


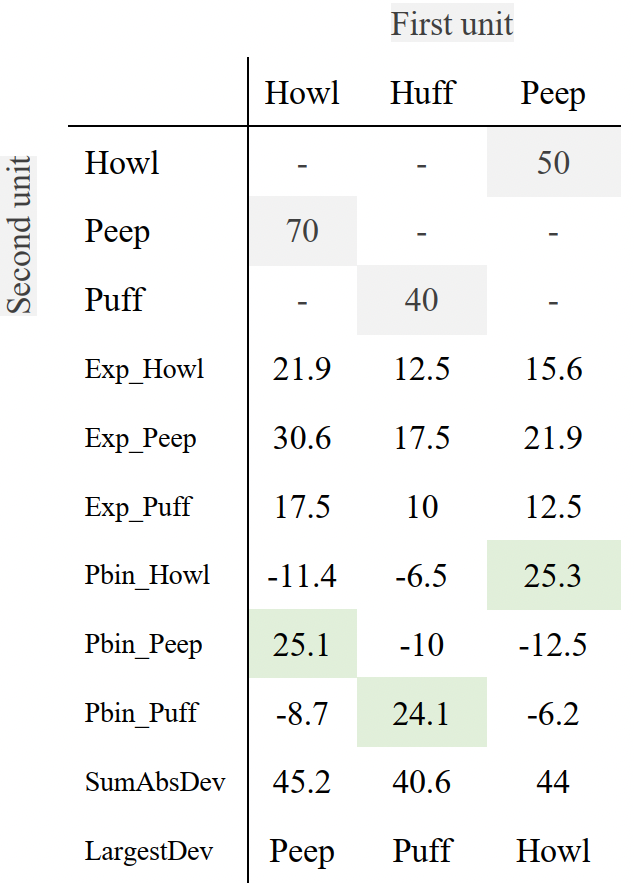
Table 8: LE (large-exclusive) data set MDCA matrix:


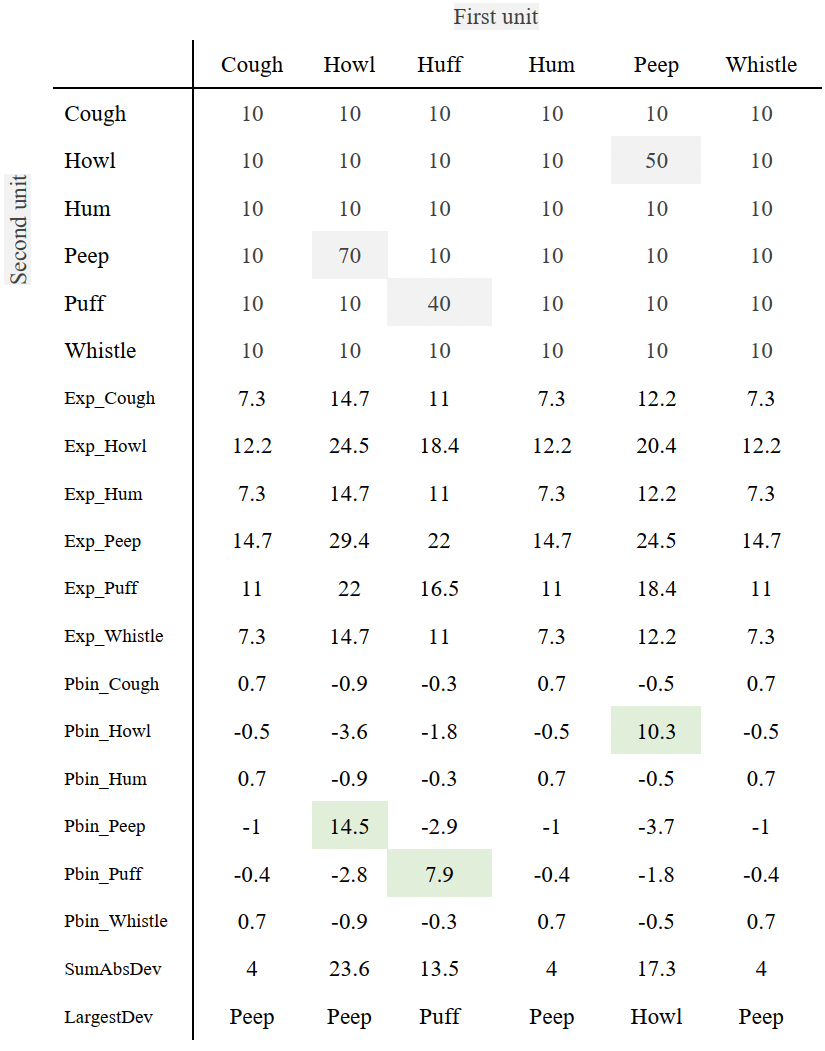
Table 9: LR (large-recombinations) data set MDCA matrix:

**Appendix III: MICA tables**

Table 10: SE (small-exclusive) data set MICA matrix:


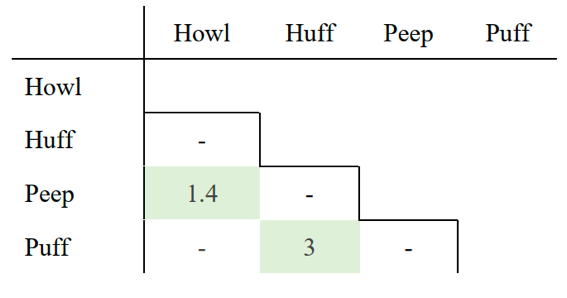


Table 11: SR (small-recombinations) data set MICA matrix:


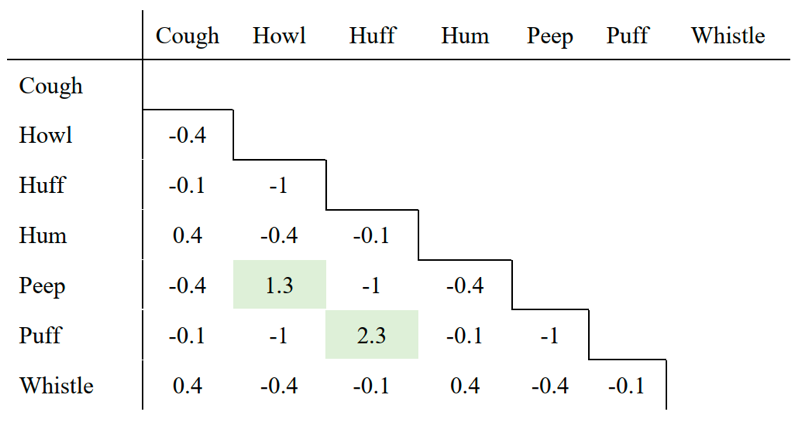


Table 12: LE (large-exclusive) data set MICA matrix:


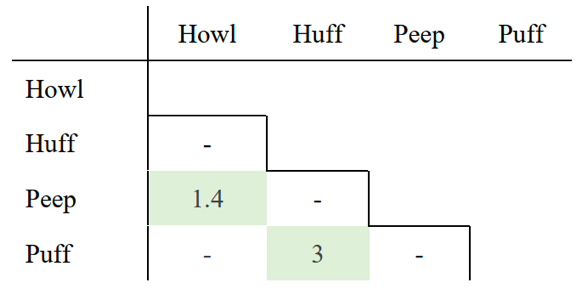


Table 13: LR (large-recombinations) data set MICA matrix:


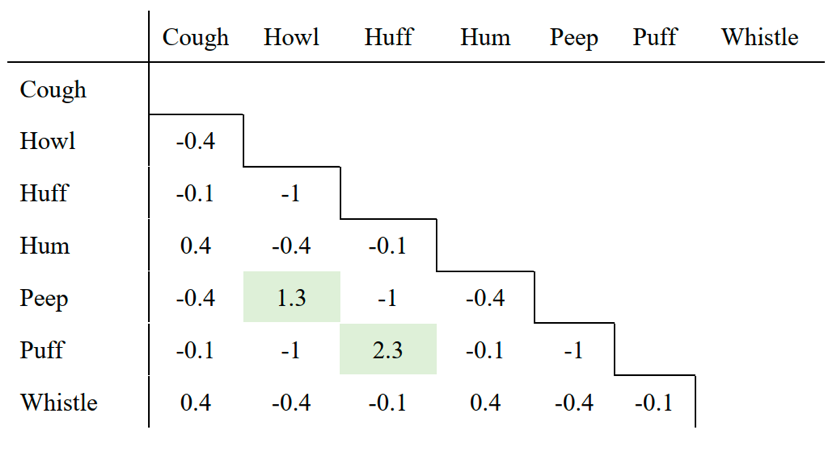


**Appendix IV: R code**

#Gries, Stefan Th. 2007. Coll.analysis 3.5. A script for R to compute collostructional analyses.

> RUN :

source("﻿https://gitlab.uzh.ch/alexandra.bosshard/call-ocation/-/raw/master/coll.analysis3.2_calls.txt")

##MDCA

> # PRESS <Enter> #

> # PRESS <Enter> #

> 2                ## (2 chooses MDCA)

> 2                ## (2 chooses 3+ categories → one-tailed exact binomial test)

> 3                ## (Choosing 3 decimals)

> # PRESS <Enter> and CHOOSE FILE ## (here “input_file_name_of_bigram_list.txt”) #

> 1                ## (1 orders output alphabetically)

> # PRESS <Enter> and CHOOSE FILE ## (here: “outcome_file_name_for_MDCA.txt”) #

##MICA

> # PRESS <Enter> #

> # PRESS <Enter> #

> 1                ## (1 chooses collocational/collexeme analysis)

> FREQ_WORD_with_CALL     ## (CALL is the investigated call unit)

> 32                 ## (Size of the whole corpus, here for SE data set)

> 4                 ## (Number of constructions comprising the call unit investigated)

> 3                ## (3 chooses MICA)

> 1                ## (1 orders output alphabetically)

> 3                ## (Choosing 3 decimals)

> # PRESS <Enter> and CHOOSE FILE (here “input_file_with_investigated_call_matrix.txt”) #

> # PRESS <Enter> and CHOOSE FILE (here: “outcome_file_name_for_MICA.txt”) #

**Appendix V: Raw input files MDCA**

**Small-Exclusive:**

Calls                 Co_Calls

Howl                Peep

Howl                Peep

Howl                Peep

Howl                Peep

Howl                Peep

Howl                Peep

Howl                Peep

Huff                 Puff

Huff                 Puff

Huff                 Puff

Huff                 Puff

Peep                Howl

Peep                Howl

Peep                Howl

Peep                Howl

Peep                Howl

**Small-Recombination:**

Calls                 Co_Calls

Cough              Cough

Cough              Howl

Cough              Hum

Cough              Peep

Cough              Puff

Cough              Whistle

Howl                Cough

Howl                Howl

Howl                Hum

Howl                Peep

Howl                Peep

Howl                Peep

Howl                Peep

Howl                Peep

Howl                Peep

Howl                Peep

Howl                Puff

Howl                Whistle

Huff                 Cough

Huff                 Howl

Huff                 Hum

Huff                 Peep

Huff                 Puff

Huff                 Puff

Huff                 Puff

Huff                 Puff

Huff                 Whistle

Hum                Cough

Hum                Howl

Hum                Hum

Hum                Peep

Hum                Puff

Hum                Whistle

Peep                Cough

Peep                Howl

Peep                Howl

Peep                Howl

Peep                Howl

Peep                Howl

Peep                Hum

Peep                Peep

Peep                Puff

Peep                Whistle

Whistle            Cough

Whistle            Howl

Whistle            Hum

Whistle            Peep

Whistle            Puff

Whistle            Whistle

**Large-Exclusive:**

Calls                 Co_Calls

Howl                Peep

Howl                Peep

Howl                Peep

Howl                Peep

Howl                Peep

Howl                Peep

Howl                Peep

Howl                Peep

Howl                Peep

Howl                Peep

Howl                Peep

Howl                Peep

Howl                Peep

Howl                Peep

Howl                Peep

Howl                Peep

Howl                Peep

Howl                Peep

Howl                Peep

Howl                Peep

Howl                Peep

Howl                Peep

Howl                Peep

Howl                Peep

Howl                Peep

Howl                Peep

Howl                Peep

Howl                Peep

Howl                Peep

Howl                Peep

Howl                Peep

Howl                Peep

Howl                Peep

Howl                Peep

Howl                Peep

Howl                Peep

Howl                Peep

Howl                Peep

Howl                Peep

Howl                Peep

Howl                Peep

Howl                Peep

Howl                Peep

Howl                Peep

Howl                Peep

Howl                Peep

Howl                Peep

Howl                Peep

Howl                Peep

Howl                Peep

Howl                Peep

Howl                Peep

Howl                Peep

Howl                Peep

Howl                Peep

Howl                Peep

Howl                Peep

Howl                Peep

Howl                Peep

Howl                Peep

Howl                Peep

Howl                Peep

Howl                Peep

Howl                Peep

Howl                Peep

Howl                Peep

Howl                Peep

Howl                Peep

Howl                Peep

Howl                Peep

Huff                 Puff

Huff                 Puff

Huff                 Puff

Huff                 Puff

Huff                 Puff

Huff                 Puff

Huff                 Puff

Huff                 Puff

Huff                 Puff

Huff                 Puff

Huff                 Puff

Huff                 Puff

Huff                 Puff

Huff                 Puff

Huff                 Puff

Huff                 Puff

Huff                 Puff

Huff                 Puff

Huff                 Puff

Huff                 Puff

Huff                 Puff

Huff                 Puff

Huff                 Puff

Huff                 Puff

Huff                 Puff

Huff                 Puff

Huff                 Puff

Huff                 Puff

Huff                 Puff

Huff                 Puff

Huff                 Puff

Huff                 Puff

Huff                 Puff

Huff                 Puff

Huff                 Puff

Huff                 Puff

Huff                 Puff

Huff                 Puff

Huff                 Puff

Huff                 Puff

Peep                Howl

Peep                Howl

Peep                Howl

Peep                Howl

Peep                Howl

Peep                Howl

Peep                Howl

Peep                Howl

Peep                Howl

Peep                Howl

Peep                Howl

Peep                Howl

Peep                Howl

Peep                Howl

Peep                Howl

Peep                Howl

Peep                Howl

Peep                Howl

Peep                Howl

Peep                Howl

Peep                Howl

Peep                Howl

Peep                Howl

Peep                Howl

Peep                Howl

Peep                Howl

Peep                Howl

Peep                Howl

Peep                Howl

Peep                Howl

Peep                Howl

Peep                Howl

Peep                Howl

Peep                Howl

Peep                Howl

Peep                Howl

Peep                Howl

Peep                Howl

Peep                Howl

Peep                Howl

Peep                Howl

Peep                Howl

Peep                Howl

Peep                Howl

Peep                Howl

Peep                Howl

Peep                Howl

Peep                Howl

Peep                Howl

Peep                Howl

**Large-Recombination:**

Calls                 Co_Calls

Cough              Cough

Cough              Cough

Cough              Cough

Cough              Cough

Cough              Cough

Cough              Cough

Cough              Cough

Cough              Cough

Cough              Cough

Cough              Cough

Cough              Howl

Cough              Howl

Cough              Howl

Cough              Howl

Cough              Howl

Cough              Howl

Cough              Howl

Cough              Howl

Cough              Howl

Cough              Howl

Cough              Hum

Cough              Hum

Cough              Hum

Cough              Hum

Cough              Hum

Cough              Hum

Cough              Hum

Cough              Hum

Cough              Hum

Cough              Hum

Cough              Peep

Cough              Peep

Cough              Peep

Cough              Peep

Cough              Peep

Cough              Peep

Cough              Peep

Cough              Peep

Cough              Peep

Cough              Peep

Cough              Puff

Cough              Puff

Cough              Puff

Cough              Puff

Cough              Puff

Cough              Puff

Cough              Puff

Cough              Puff

Cough              Puff

Cough              Puff

Cough              Whistle

Cough              Whistle

Cough              Whistle

Cough              Whistle

Cough              Whistle

Cough              Whistle

Cough              Whistle

Cough              Whistle

Cough              Whistle

Cough              Whistle

Howl                Cough

Howl                Cough

Howl                Cough

Howl                Cough

Howl                Cough

Howl                Cough

Howl                Cough

Howl                Cough

Howl                Cough

Howl                Cough

Howl                Howl

Howl                Howl

Howl                Howl

Howl                Howl

Howl                Howl

Howl                Howl

Howl                Howl

Howl                Howl

Howl                Howl

Howl                Howl

Howl                Hum

Howl                Hum

Howl                Hum

Howl                Hum

Howl                Hum

Howl                Hum

Howl                Hum

Howl                Hum

Howl                Hum

Howl                Hum

Howl                Peep

Howl                Peep

Howl                Peep

Howl                Peep

Howl                Peep

Howl                Peep

Howl                Peep

Howl                Peep

Howl                Peep

Howl                Peep

Howl                Peep

Howl                Peep

Howl                Peep

Howl                Peep

Howl                Peep

Howl                Peep

Howl                Peep

Howl                Peep

Howl                Peep

Howl                Peep

Howl                Peep

Howl                Peep

Howl                Peep

Howl                Peep

Howl                Peep

Howl                Peep

Howl                Peep

Howl                Peep

Howl                Peep

Howl                Peep

Howl                Peep

Howl                Peep

Howl                Peep

Howl                Peep

Howl                Peep

Howl                Peep

Howl                Peep

Howl                Peep

Howl                Peep

Howl                Peep

Howl                Peep

Howl                Peep

Howl                Peep

Howl                Peep

Howl                Peep

Howl                Peep

Howl                Peep

Howl                Peep

Howl                Peep

Howl                Peep

Howl                Peep

Howl                Peep

Howl                Peep

Howl                Peep

Howl                Peep

Howl                Peep

Howl                Peep

Howl                Peep

Howl                Peep

Howl                Peep

Howl                Peep

Howl                Peep

Howl                Peep

Howl                Peep

Howl                Peep

Howl                Peep

Howl                Peep

Howl                Peep

Howl                Peep

Howl                Peep

Howl                Puff

Howl                Puff

Howl                Puff

Howl                Puff

Howl                Puff

Howl                Puff

Howl                Puff

Howl                Puff

Howl                Puff

Howl                Puff

Howl                Whistle

Howl                Whistle

Howl                Whistle

Howl                Whistle

Howl                Whistle

Howl                Whistle

Howl                Whistle

Howl                Whistle

Howl                Whistle

Howl                Whistle

Huff                 Cough

Huff                 Cough

Huff                 Cough

Huff                 Cough

Huff                 Cough

Huff                 Cough

Huff                 Cough

Huff                 Cough

Huff                 Cough

Huff                 Cough

Huff                 Howl

Huff                 Howl

Huff                 Howl

Huff                 Howl

Huff                 Howl

Huff                 Howl

Huff                 Howl

Huff                 Howl

Huff                 Howl

Huff                 Howl

Huff                 Hum

Huff                 Hum

Huff                 Hum

Huff                 Hum

Huff                 Hum

Huff                 Hum

Huff                 Hum

Huff                 Hum

Huff                 Hum

Huff                 Hum

Huff                 Peep

Huff                 Peep

Huff                 Peep

Huff                 Peep

Huff                 Peep

Huff                 Peep

Huff                 Peep

Huff                 Peep

Huff                 Peep

Huff                 Peep

Huff                 Puff

Huff                 Puff

Huff                 Puff

Huff                 Puff

Huff                 Puff

Huff                 Puff

Huff                 Puff

Huff                 Puff

Huff                 Puff

Huff                 Puff

Huff                 Puff

Huff                 Puff

Huff                 Puff

Huff                 Puff

Huff                 Puff

Huff                 Puff

Huff                 Puff

Huff                 Puff

Huff                 Puff

Huff                 Puff

Huff                 Puff

Huff                 Puff

Huff                 Puff

Huff                 Puff

Huff                 Puff

Huff                 Puff

Huff                 Puff

Huff                 Puff

Huff                 Puff

Huff                 Puff

Huff                 Puff

Huff                 Puff

Huff                 Puff

Huff                 Puff

Huff                 Puff

Huff                 Puff

Huff                 Puff

Huff                 Puff

Huff                 Puff

Huff                 Puff

Huff                 Whistle

Huff                 Whistle

Huff                 Whistle

Huff                 Whistle

Huff                 Whistle

Huff                 Whistle

Huff                 Whistle

Huff                 Whistle

Huff                 Whistle

Huff                 Whistle

Hum                Cough

Hum                Cough

Hum                Cough

Hum                Cough

Hum                Cough

Hum                Cough

Hum                Cough

Hum                Cough

Hum                Cough

Hum                Cough

Hum                Howl

Hum                Howl

Hum                Howl

Hum                Howl

Hum                Howl

Hum                Howl

Hum                Howl

Hum                Howl

Hum                Howl

Hum                Howl

Hum                Hum

Hum                Hum

Hum                Hum

Hum                Hum

Hum                Hum

Hum                Hum

Hum                Hum

Hum                Hum

Hum                Hum

Hum                Hum

Hum                Peep

Hum                Peep

Hum                Peep

Hum                Peep

Hum                Peep

Hum                Peep

Hum                Peep

Hum                Peep

Hum                Peep

Hum                Peep

Hum                Puff

Hum                Puff

Hum                Puff

Hum                Puff

Hum                Puff

Hum                Puff

Hum                Puff

Hum                Puff

Hum                Puff

Hum                Puff

Hum                Whistle

Hum                Whistle

Hum                Whistle

Hum                Whistle

Hum                Whistle

Hum                Whistle

Hum                Whistle

Hum                Whistle

Hum                Whistle

Hum                Whistle

Peep                Cough

Peep                Cough

Peep                Cough

Peep                Cough

Peep                Cough

Peep                Cough

Peep                Cough

Peep                Cough

Peep                Cough

Peep                Cough

Peep                Howl

Peep                Howl

Peep                Howl

Peep                Howl

Peep                Howl

Peep                Howl

Peep                Howl

Peep                Howl

Peep                Howl

Peep                Howl

Peep                Howl

Peep                Howl

Peep                Howl

Peep                Howl

Peep                Howl

Peep                Howl

Peep                Howl

Peep                Howl

Peep                Howl

Peep                Howl

Peep                Howl

Peep                Howl

Peep                Howl

Peep                Howl

Peep                Howl

Peep                Howl

Peep                Howl

Peep                Howl

Peep                Howl

Peep                Howl

Peep                Howl

Peep                Howl

Peep                Howl

Peep                Howl

Peep                Howl

Peep                Howl

Peep                Howl

Peep                Howl

Peep                Howl

Peep                Howl

Peep                Howl

Peep                Howl

Peep                Howl

Peep                Howl

Peep                Howl

Peep                Howl

Peep                Howl

Peep                Howl

Peep                Howl

Peep                Howl

Peep                Hum

Peep                Hum

Peep                Hum

Peep                Hum

Peep                Hum

Peep                Hum

Peep                Hum

Peep                Hum

Peep                Hum

Peep                Hum

Peep                Peep

Peep                Peep

Peep                Peep

Peep                Peep

Peep                Peep

Peep                Peep

Peep                Peep

Peep                Peep

Peep                Peep

Peep                Peep

Peep                Puff

Peep                Puff

Peep                Puff

Peep                Puff

Peep                Puff

Peep                Puff

Peep                Puff

Peep                Puff

Peep                Puff

Peep                Puff

Peep                Whistle

Peep                Whistle

Peep                Whistle

Peep                Whistle

Peep                Whistle

Peep                Whistle

Peep                Whistle

Peep                Whistle

Peep                Whistle

Peep                Whistle

Whistle            Cough

Whistle            Cough

Whistle            Cough

Whistle            Cough

Whistle            Cough

Whistle            Cough

Whistle            Cough

Whistle            Cough

Whistle            Cough

Whistle            Cough

Whistle            Howl

Whistle            Howl

Whistle            Howl

Whistle            Howl

Whistle            Howl

Whistle            Howl

Whistle            Howl

Whistle            Howl

Whistle            Howl

Whistle            Howl

Whistle            Hum

Whistle            Hum

Whistle            Hum

Whistle            Hum

Whistle            Hum

Whistle            Hum

Whistle            Hum

Whistle            Hum

Whistle            Hum

Whistle            Hum

Whistle            Peep

Whistle            Peep

Whistle            Peep

Whistle            Peep

Whistle            Peep

Whistle            Peep

Whistle            Peep

Whistle            Peep

Whistle            Peep

Whistle            Peep

Whistle            Puff

Whistle            Puff

Whistle            Puff

Whistle            Puff

Whistle            Puff

Whistle            Puff

Whistle            Puff

Whistle            Puff

Whistle            Puff

Whistle            Puff

Whistle            Whistle

Whistle            Whistle

Whistle            Whistle

Whistle            Whistle

Whistle            Whistle

Whistle            Whistle

Whistle            Whistle

Whistle            Whistle

Whistle            Whistle

Whistle            Whistle

**Appendix VI: Raw input files MICA**

**Small-Exclusive:**

***Howl-Peep matrix:***

WORD FREQ_WORD_in_CORPUS FREQ_WORD_with_howl

Bark 0                    0

Cough        0        0

Cry        0                    0

Howl        12                    0

Huff        4                    0

Hum        0                    0

Peep        12                    12

Puff        4                    0

Twitter        0                    0

Whistle        0                    0

***Huff-Puff matrix:***

WORD        FREQ_WORD_in_CORPUS FREQ_WORD_with_huff

Bark        0                    0

Cough        0                    0

Cry        0                    0

Howl        12                    0

Huff        4                    0

Hum        0                    0

Peep        12                    0

Puff        4                        4

Twitter        0                    0

Whistle        0                    0

**Small-Recombination:**

***Howl-Peep matrix:***

WORD        FREQ_WORD_in_CORPUS FREQ_WORD_with_howl

Bark        0                    0

Cough        12                    2

Cry        0                    0

Howl        12                    1

Huff        9                    1

Hum        12                    2

Peep        22                    12

Puff        9                    1

Twitter        0                    0

Whistle        12                    2

***Huff-Puff matrix:***

WORD        FREQ_WORD_in_CORPUS   FREQ_WORD_with_huff

Bark        0                    0

Cough        12                    1

Cry        0                    0

Howl        22                    1

Huff        9                    0

Hum        12                    1

Peep        22                    1

Puff        9                       4

Twitter        0                    0

Whistle        12                    1

**Large-Exclusive:**

***Howl-Peep matrix:***

WORD FREQ_WORD_in_CORPUS  FREQ_WORD_with_howl

Bark        0                    0

Cough        0                    0

Cry        0                    0

Howl        120                    0

Huff        40                    0

Hum        0                    0

Peep        120                    120

Puff        40                    0

Twitter        0                    0

Whistle        0                    0

***Huff-Puff matrix:***

WORD        FREQ_WORD_in_CORPUS   FREQ_WORD_with_huff

Puff        40                        40

Bark        0                    0

Cough        0                    0

Cry        0                    0

Howl        120                    0

Huff        40                    0

Hum        0                    0

Peep        120                    0

Twitter        0                    0

Whistle        0                    0

**Large-Recombination:**

***Howl-Peep matrix:***

WORD        FREQ_WORD_in_CORPUS  FREQ_WORD_with_howl

Bark        0                    0

Cough        120                    20

Cry        0                    0

Howl        120                    10

Huff        90                    10

Hum        120                    20

Peep        220                    120

Puff        90                    10

Twitter        0                    0

Whistle        120                    20

***Huff-Puff matrix:***

WORD      FREQ_WORD_in_CORPUS   FREQ_WORD_with_huff

Puff        90                        40

Bark        0                    0

Cough        120                    10

Cry        0                    0

Howl        220                    10

Huff        90                    0

Hum        120                    10

Peep        220                    10

Twitter        0                    0

Whistle        120                    10
